# Nonsyndromic Agminated Basal Cell Carcinomas: Diagnostic and Therapeutic Conundrum Case Report

**DOI:** 10.2196/87200

**Published:** 2026-11-13

**Authors:** Sarita Pattisam, Kelly Kimball, Austin Cusick, Angela Casey

**Affiliations:** 1 College of Medicine, Ohio University Heritage College of Osteopathic Medicine, Columbus, OH, United States; 2 Department of Dermatology, OhioHealth, 3535 Olentangy River Rd, Columbus, OH, 43214, United States, 1 4195128350; 3 Department of Dermatology, Lehigh Valley Hospital, Allentown, PA, United States; 4 Department of Dermatology, Center for Surgical Dermatology, Westerville, OH, United States

**Keywords:** basal cell carcinoma, nevoid basal cell carcinoma syndrome, Mohs micrographic surgery, Gorlin-Goltz syndrome, agminated basal cell carcinoma

## Abstract

Basal cell carcinoma (BCC) is the most common cutaneous malignancy, with multiple localized lesions typically linked to genodermatoses or environmental exposures. Rarely, nonsyndromic BCCs (NSBCCs) may be present in an agminated pattern without identifiable genetic or environmental risk factors. We present two patients with numerous agminated BCCs without syndromic features: one with focal distribution of BCCs along the frontal scalp/hairline, and the other with unilateral distribution of BCCs on the right side of the face. Patient 1, a woman aged 67 years, developed 22 BCCs over 24 years, 19 localized to the frontal scalp. Patient 2, a man aged 78 years, developed 44 BCCs over 11 years, predominantly on the right face, with a history of adolescent full-face radiation, representing the highest number of nonsyndromic agminated BCCs reported to date. We report these two cases of nonsyndromic agminated BCCs treated with surgical and destructive modalities. Their strikingly focal distribution raises the possibility of localized genetic or environmental factors, highlighting the need for further studies to clarify the mechanisms underlying nonsyndromic agminated BCC and determine whether it represents a distinct entity.

## Introduction

Basal cell carcinoma (BCC) is the most common cutaneous malignancy treated by dermatologists [1]. Most BCCs are sporadic; however, certain genodermatoses may predispose patients to developing BCCs at an earlier age and in greater numbers [2]. Examples of such genodermatoses include nevoid basal cell carcinoma syndrome (NBCCS), also known as Gorlin-Goltz syndrome, Bazex-Dupré-Christol syndrome, xeroderma pigmentosum, and Rombo syndrome [2]. These diseases are typically associated with a constellation of extracutaneous symptoms that cue dermatologists to make the appropriate diagnosis. We define agminated BCC as the development of multiple, histologically confirmed basal cell carcinomas clustered within a discrete, localized region of skin. Unlike many previously reported agminated presentations, which describe a cluster of lesions arising over a short, discrete time interval, the two patients we present developed their clustered lesions progressively over more than a decade, raising the question of whether a temporal criterion should be incorporated into the definition. Most sporadic BCCs have a propensity for skin with a history of significant sun exposure/sunburns over the individual’s lifetime. Other factors, such as radiation, chemical exposure to carcinogens such as arsenic, and thermal burns, may increase the risk of BCCs in a specific area of the skin. Rarely do sporadic BCCs, in the absence of associated genetic mutations or injury/exposure of the affected skin, aggregate in a specific location, sparing other body parts.

## Case Report

### Case 1

A female aged 67 years with a past medical history significant for a port wine stain of the posterior neck presented with 22 BCCs over 24 years (2000‐2024). All BCCs were diagnosed by an external dermatopathologist and confirmed in the setting of Mohs micrographic surgery (MMS) by the surgeon. Of these, 19 were localized to the frontal scalp region. The lesion distribution is visualized in Figure 1, and a clinical photograph depicting representative lesions is shown in Figure 2. The patient reported no personal history of immunosuppression and denied any family history of skin cancer. She also denied any history of significant or unusual sun exposure patterns/sunburns, radiation, trauma, injury, or contact with chemicals involving the affected areas. A complete cutaneous examination did not reveal any other significant findings of the skin. Genetic syndromes associated with basal cell carcinoma development were excluded as the patient lacked associated stigmata. She was treated with a combination of electrodesiccation and curettage (ED&C), MMS, and standard surgical excision.

**Figure 1.**
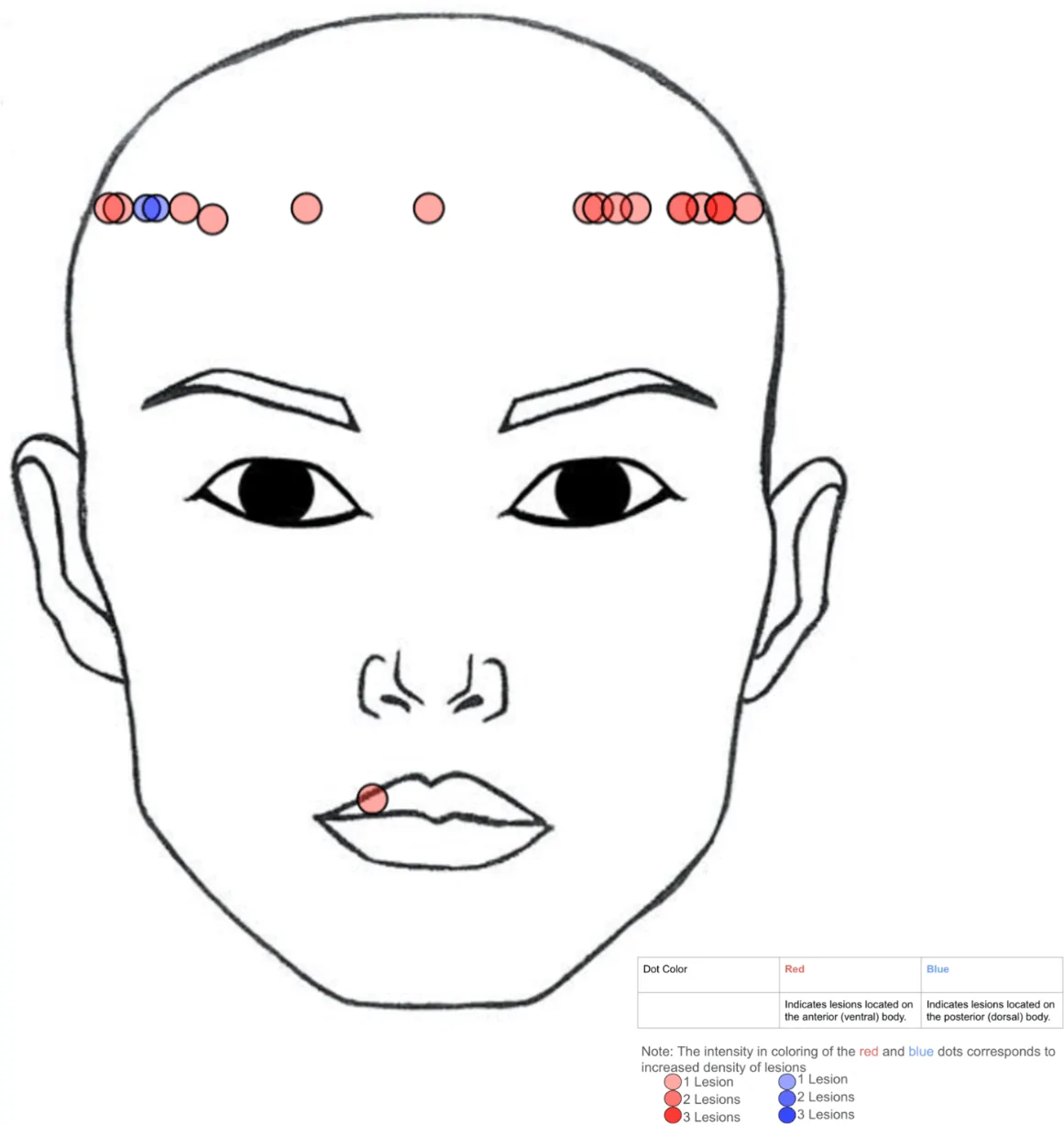
Location of basal cell carcinomas on patient 1. The red dots denote lesions on the ventral aspect of the body, while the blue dots denote lesions on the dorsal aspect.

**Figure 2.**
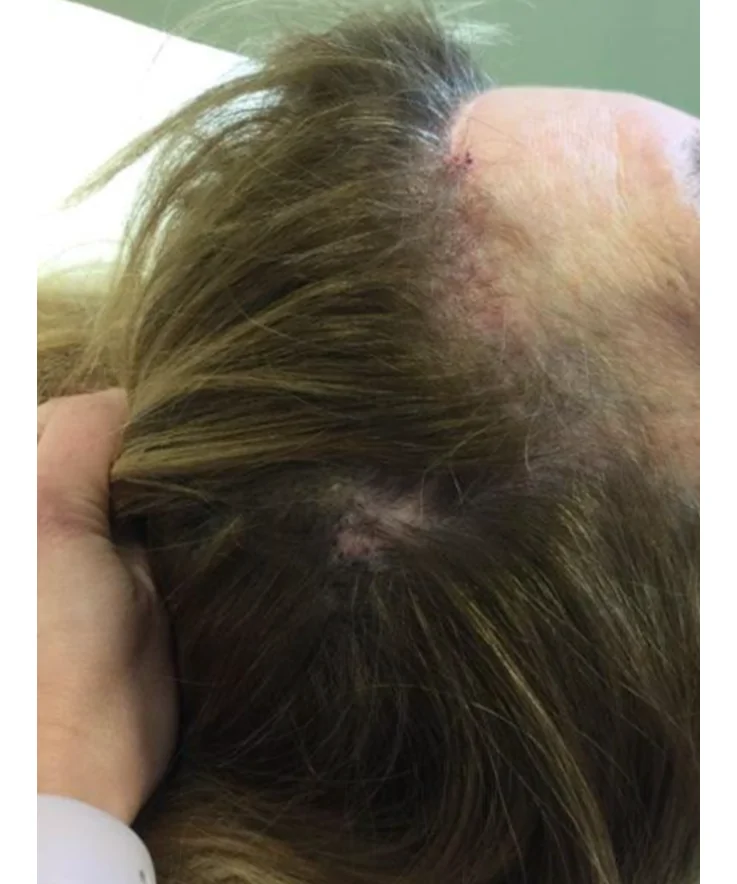
Clinical image depicting representative lesions along patient 1’s hairline.

### Case 2

A male aged 78 years presented with 44 BCCs over 11 years (2013‐2024). All BCCs were diagnosed by an external dermatopathologist and confirmed in the setting of MMS by the surgeon. Of the 44 BCCs, 24 (54%) occurred on the right side of the face, and 20 of these were considered agminated based on their clustered distribution within the right perioral and buccal area. The lesion distribution is visualized in Figure 3. His history is significant for radiation treatment for acne to his entire face over a 3-month period when he was a teenager. Notably, although the patient’s entire face had previously been irradiated for acne, his facial BCCs demonstrated a striking unilateral distribution, occurring almost exclusively on the right side. He reported no history of immunosuppression and denied any family history of genetic syndromes associated with BCCs. He also denied any history of significant or unusual sun exposure patterns/sunburns, trauma, injury, or contact with chemicals involving the affected areas. A complete cutaneous examination did not reveal other significant changes on the skin. Genetic syndromes associated with BCCs were excluded as the patient lacked associated stigmata. He was treated with a combination of ED&C, MMS, and standard surgical excision. A clinical photograph depicting representative lesions is shown in Figure 4, and a histopathologic examination of one of these lesions is shown in Figure 5.

**Figure 3.**
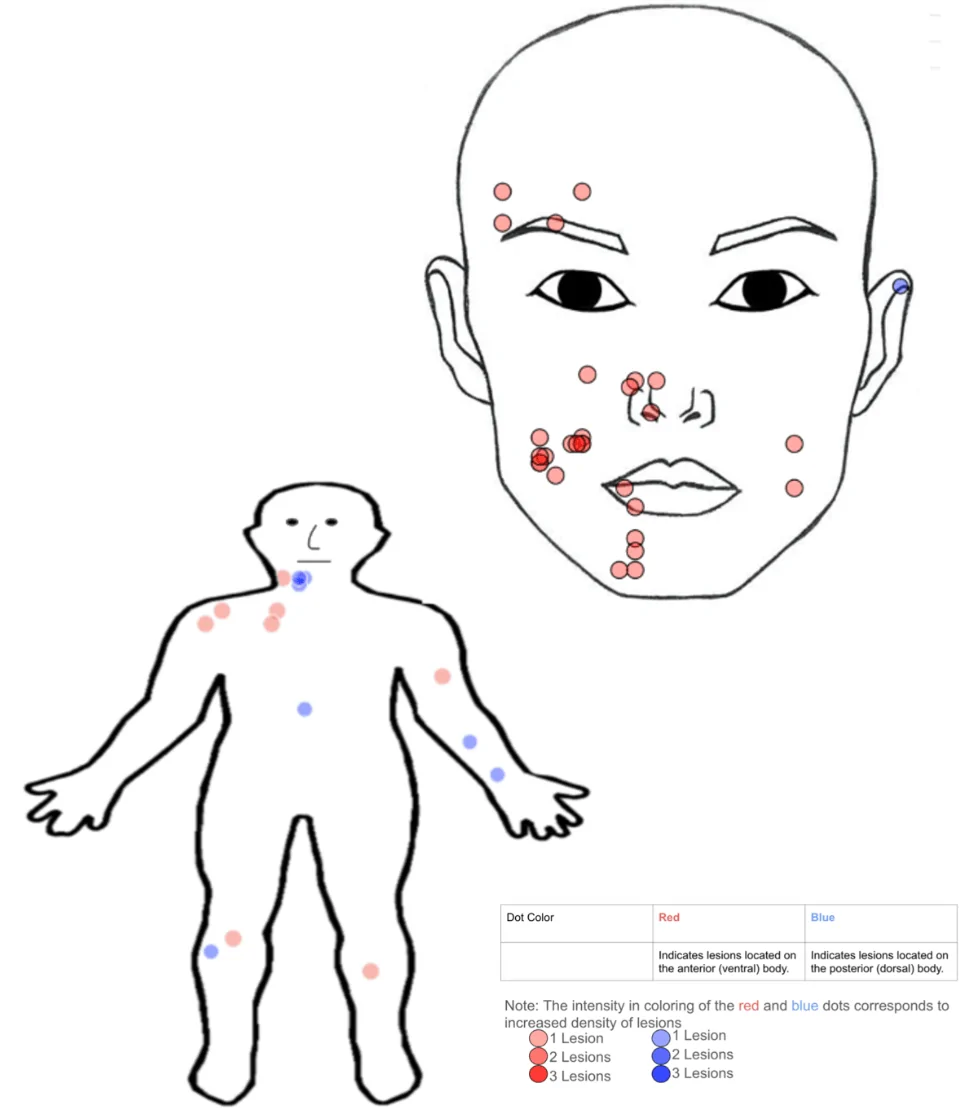
Location of basal cell carcinomas on patient 2. The red dots denote lesions on the ventral aspect of the body, while the blue dots denote lesions on the dorsal aspect.

**Figure 4.**
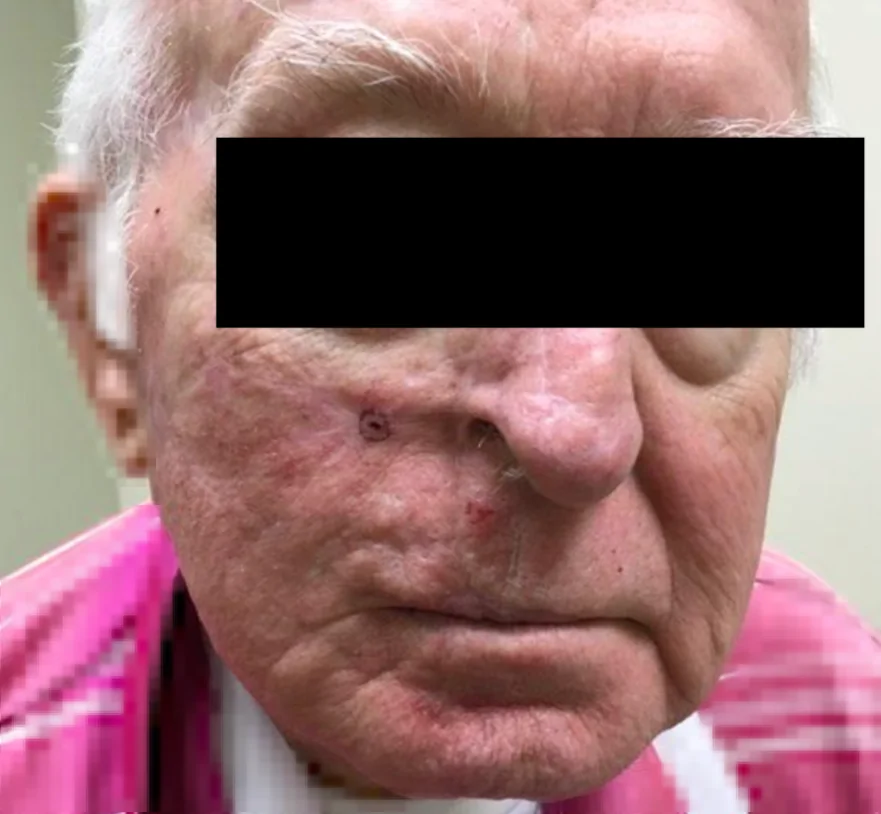
Clinical image depicting representative lesions on the right side of patient 2’s face. Note the appreciable disfiguration and scarring compared to the left side.

**Figure 5.**
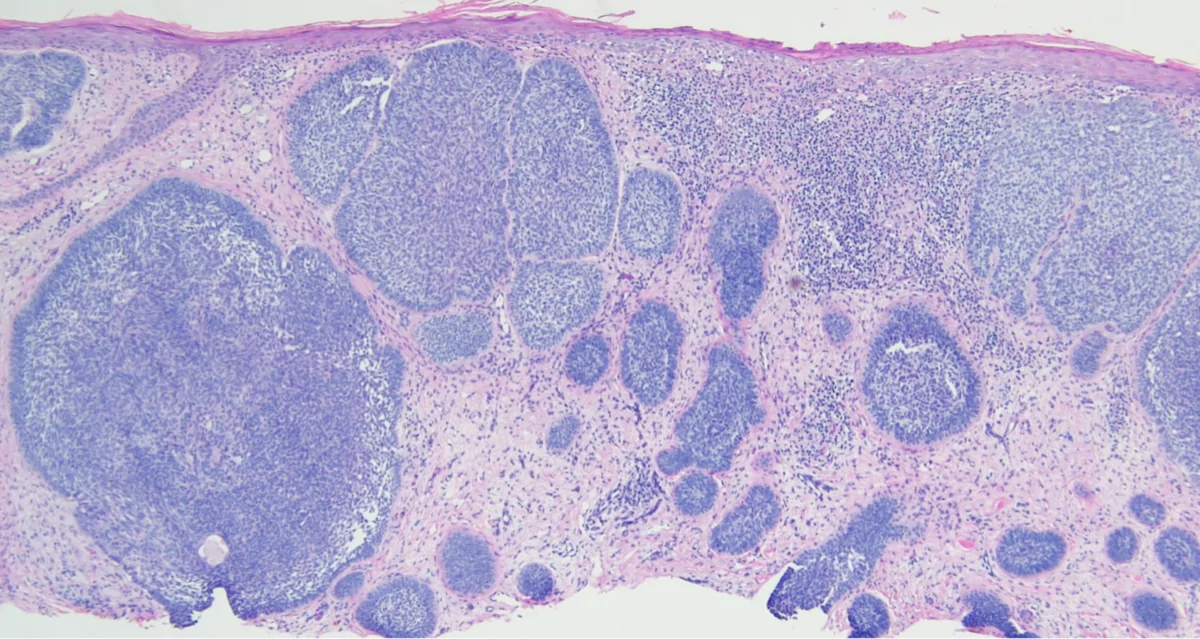
Histopathology of one of the numerous lesions in patient 2.

### Ethical Considerations

The authors obtained written consent from patients for their photographs and medical information to be published in print and online and with the understanding that this information may be publicly available. Patient consent forms were not provided to the journal but are retained by the authors.

## Discussion

Nonsyndromic development of numerous agminated BCCs is rarely reported and presents diagnostic and therapeutic challenges. Postzygotic somatic mutations affecting the sonic hedgehog (SHH) pathway, including protein patched homolog 1 (PTCH1), protein patched homolog 1 (PTCH2), suppressor of fused (SUFU), and smoothened (SMO), have been hypothesized in localized agminated BCCs, although this remains unconfirmed [3,4]. Patient 2 had prior full-face radiation for acne; however, this bilateral exposure does not fully explain the predominantly right-sided tumor burden, raising the possibility of additional localized susceptibility such as cutaneous mosaicism. Gene sequencing of affected skin was not performed, and mosaicism therefore remains a hypothesis. Although cutaneous mosaicism classically follows the lines of Blaschko, neither patient demonstrated a definitive distribution: patient 2’s lesions could span multiple right facial Blaschko lines, whereas patient 1’s horizontal distribution did not follow the longitudinal Blaschko lines of the forehead and frontal scalp. Systemic genetic testing was not pursued because neither patient exhibited other features of a BCC-associated genetic syndrome. Accordingly, both were diagnosed with nonsyndromic agminated BCC as a diagnosis of exclusion.

Previously reported cases identified through our narrative review are summarized in the table found in Multimedia Appendix 1. Because a systematic search was not performed, additional published, nonindexed, or unpublished cases may have been missed. Patient 2 represents, to our knowledge, the highest reported number of nonsyndromic agminated BCCs to date.

Treatment is challenging when numerous lesions involve a confined anatomic region. Although surgical excision and MMS offer high cure rates, repeated procedures may become impractical because of surgical burden and potential disfigurement [4]. Alternative approaches reported include topical 5-fluorouracil, imiquimod, carbon dioxide laser ablation, and photodynamic therapy [4]. In nevoid BCC syndrome, photodynamic therapy and SHH inhibitors have reduced development of new BCCs, suggesting potential relevance for patients with extensive nonsyndromic disease [5,6]. Topical sirolimus has also shown benefit in pediatric patients with NSBCC [7]. Acitretin and nicotinamide are guideline-supported chemopreventive options for patients at increased risk of nonmelanoma skin cancer, although their use in nonsyndromic agminated BCC remains largely unreported [8,9]. Ultimately, there is sufficient evidence that employing chemopreventive strategies and SHH inhibitors could greatly benefit patients with NSBCC experiencing surgical burnout [10].

## Conclusions

We present two patients with nonsyndromic agminated BCCs successfully treated with surgical and destructive techniques. The focal clustering of numerous BCCs raises the possibility of underlying mosaicism or unrecognized environmental factors. Nonsyndromic agminated BCC may therefore represent a diagnosis of exclusion characterized by focal, nonlinear clustering. Further studies are needed to clarify its molecular basis and determine whether it represents a distinct disease entity.

## Supplementary material

10.2196/87200 Multimedia Appendix 1 Review of reported cases of nonsyndromic basal cell carcinomas (1-4, 9).

## Abbreviations

BCC: basal cell carcinoma
ED&C: electrodesiccation and curettage
MMS: Mohs micrographic surgery
NSBCC: nonsyndromic basal cell carcinoma
PTCH1: Patched 1
PTCH2: Patched 2
SHH: sonic hedgehog
SMO: smoothened
SUFU: suppressor of fused
