# Supplementary material for: Nonsyndromic Agminated Basal Cell Carcinomas: Diagnostic and Therapeutic Conundrum Case Report
Source: JMIR Dermatol. 2026 Nov 13;9:e87200. doi: 10.2196/87200 (PMC13765788; doi:10.2196/87200)
Supplement: Supplementary file 1 [file derma-v9-e87200-s001.docx]

| **Authors (year published)** | **Age, years/ Sex (M or F)** | **BCCs (*n*)** | **Predominant anatomic Location** | **Histological Type** | **Treatment** |
| --- | --- | --- | --- | --- | --- |
| Moulin et al. (1988) | 61/F | 11 | Left side of the trunk | N/A | N/A |
| Bouscarat et al. (1990) | 92/M | 30 | Left side of face, trunk, scrotum, arm and thigh | N/A | N/A |
| Lane et al. (2005) | 56/M | N/A | Left upper back | N/A | Photodynamic therapy |
| Yoshikawa et al. (2005) | 78/F | 3 | Right side of abdomen/right buttock | N/A | Standard excision |
| Weiss et al. (2015) | 61/M | N/A | Right side of face and trunk | Nodular | Standard excision, cryotherapy, and electrosurgery |
| Krajewski et al. (2020) | 55/F | 31 | Left side of trunk, extremities, neck, and behind left ear | Nodular, superficial | Standard excision, cryotherapy |
| **Our Cases** |  |  |  |  |  |
| Case 1 | 67/F | 22 | Frontal hairline and scalp (L>R) |  | Standard excision, Mohs micrographic surgery, ED&C |
| Case 2 | 78/M | 44 | Majority on right side of face | Nodular, superficial, baso-squamous, mixed | Standard excision, Mohs micrographic surgery, ED&C |

N/A = not reported/not available.
